# Supplementary material for: Association of Vaccine Confidence and Hesitancy in Three Phases of COVID-19 Vaccine Approval and Introduction in Japan
Source: Vaccines (Basel). 2022 Mar 10;10(3):423. doi: 10.3390/vaccines10030423 (PMC8954745; doi:10.3390/vaccines10030423)
Supplement: Supplementary file 1 [file vaccines-10-00423-s001.zip › vaccines-1626122-supplementary.pdf]

vaccines

MDPI

# Supplementary Materials

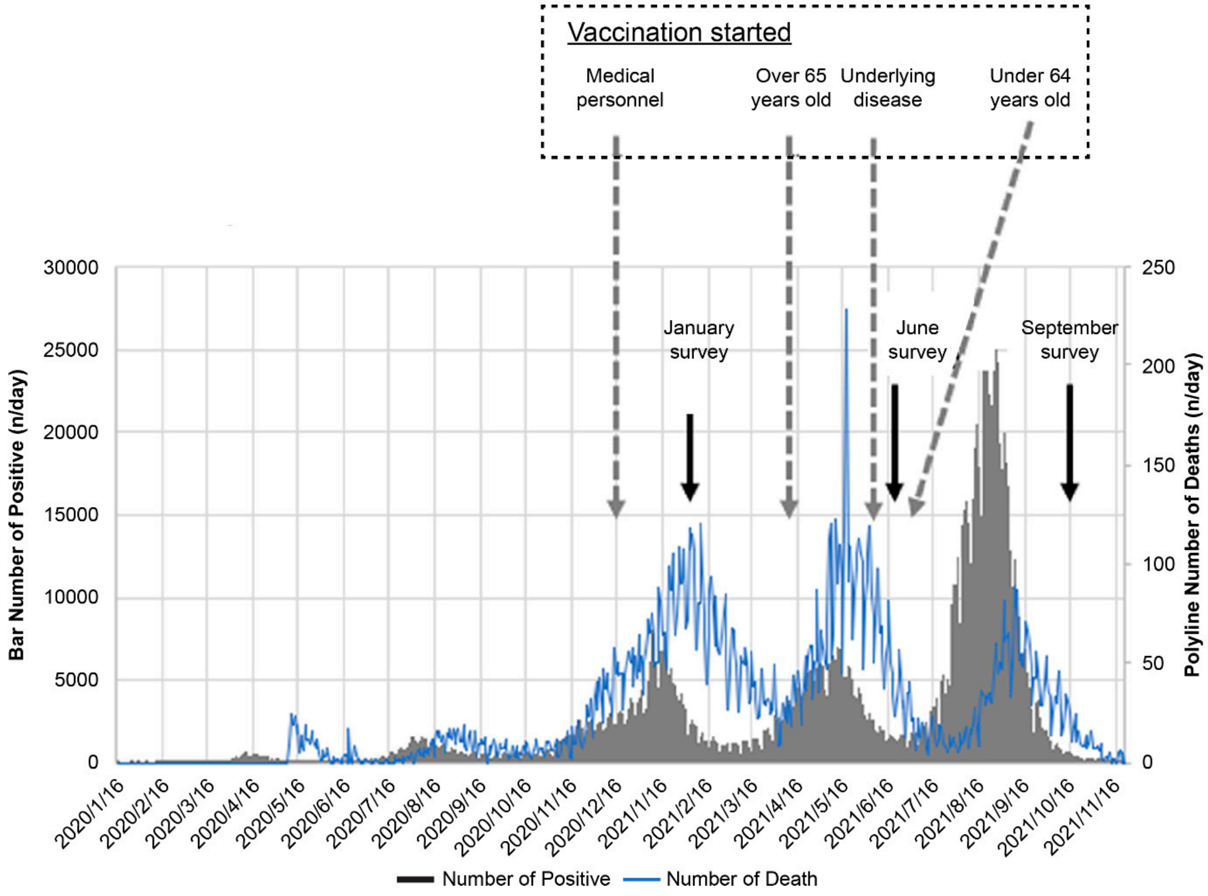


**Supplementary Figure S1.** Trends in the number of PCR test positive cases and deaths, and the timing of investigation and vaccination status. The figures were created in Excel based on the "Open data" of "Trend in the number of newly confirmed cases (daily)" and "Trends in the number of Deaths" published on the following websites. Authority: Visualizing the data: information on COVID-19. (https://covid19.mhlw.go.jp/) Access 20211122. The vaccination rate for the entire population was: as of January survey; 0%, as of June survey; 19.2%, and as of September survey; 66.5%. https://www.kantei.go.jp/jp/headline/kansensho/vaccine.html. Access 20220111

| Vaccinated at least once | Reason for not vaccinated | | |
|---|---|---|---|
| | A. Because the invitation hasn't arrived. | B. Because the appointment date has not arrived | Not A or B |
| | Not hesitancy | | Hesitancy |

**Supplementary Figure S2**. Definition of "Hesitant" at the time of the June and September surveys

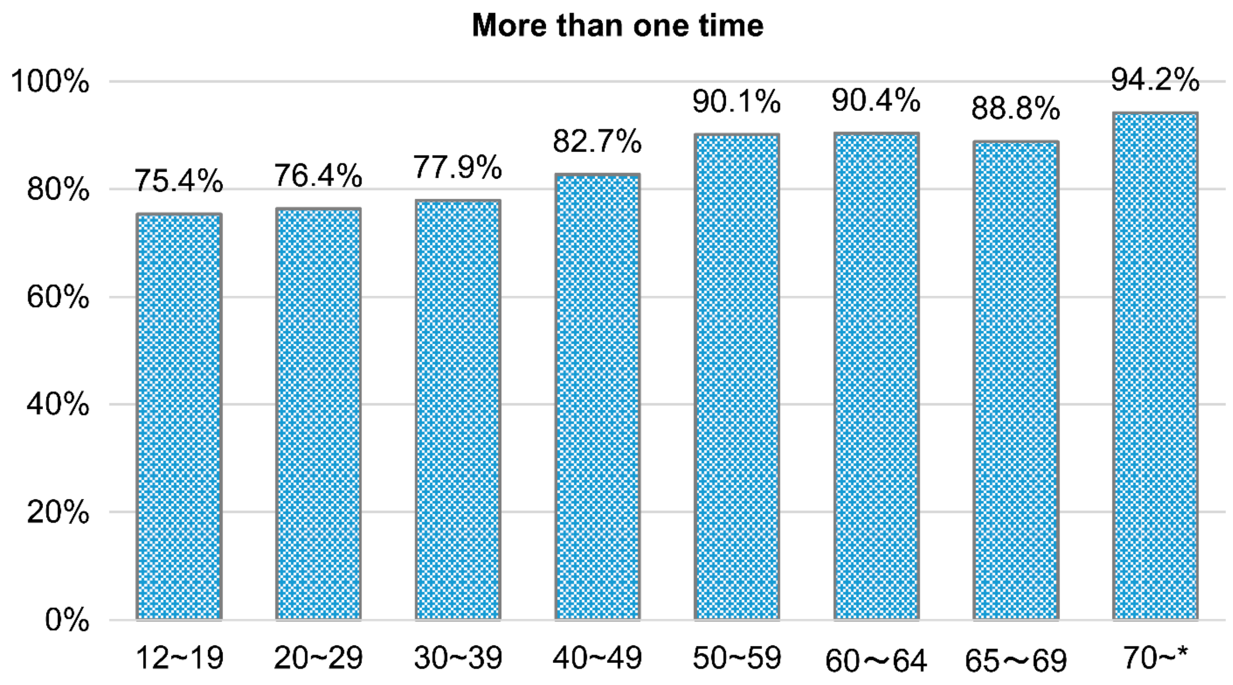


**Supplementary Figure S3**. Vaccination status by age group (one or more doses). The figure was drawn based on the results of vaccination by age group (as of December 27, 2021) published by Cabinet Public Affairs Office, Cabinet Secretariat. *"70~" is the average of the 70s, 80s, 90s, and 100s. https://www.kantei.go.jp/jp/headline/kansensho/vaccine.html. Access 20220111

**Supplementary Table S1**. Vaccine confidence and literacy items that were not significantly different between those with and without hesitancy (Age: 20-29)

| | January | | | June | | | September | | |
|---|---|---|---|---|---|---|---|---|---|
| | AOR | 95% CI | *P* value | AOR | 95% CI | *P* value | AOR | 95% CI | *P* value |
| Vaccines are important for my health | | | | | | | | | |
| Vaccines are effective | | | | | | | | | |
| Vaccines are safe | | | | | | | | | |
| My vaccination is important for the health of others in my community | | | | | | | | | |
| I am concerned about serious adverse effects of vaccines | | | | 1.03 | 0.88-1.22 | 0.685 | | | |
| New vaccines carry more risks than older vaccines | | | | 0.94 | 0.80-1.11 | 0.454 | | | |
| Serious adverse reactions may occur due to the vaccination | | | | | | | | | |
| I have difficulty getting immunized (no time, far medical institutions, etc.) | | | | | | | | | |
| I do not need vaccines for diseases that are not common anymore | 0.99 | 0.84-1.16 | 0.862 | | | | | | |

| | | | | | | | | | |
|---|---|---|---|---|---|---|---|---|---|
| We do not necessary to take voluntary vaccination | | | | | | | | | |
| I do not take vaccine, if everyone around me is immunized | | | | | | | | | |
| It is easy to obtain correct information on immunization | | | | | | | | | |
| It is easy to understand why immunization is needed. | | | | | | | | | |
| I have been able to accurately understand the vaccinations I have received | | | | | | | | | |

**Supplementary Table S2**. Vaccine confidence and literacy items that were not significantly different between those with and without hesitancy（Health care providers)

| | January | | | June | | | September | | |
|---|---|---|---|---|---|---|---|---|---|
| | AOR | 95% CI | *P* value | AOR | 95% CI | *P* value | AOR | 95% CI | *P* value |
| Vaccines are important for my health | | | | | | | | | |
| Vaccines are effective | | | | | | | | | |
| Vaccines are safe | | | | | | | | | |
| My vaccination is important for the health of others in my community | | | | | | | | | |
| I am concerned about serious adverse effects of vaccines | 1.18 | 0.94-1.48 | 0.159 | 1.02 | 0.83-1.26 | 0.850 | | | |
| New vaccines carry more risks than older vaccines | | | | | | | | | |
| Serious adverse reactions may occur due to the vaccination | | | | | | | | | |
| I have difficulty getting immunized (no time, far medical institutions, etc.) | 1.16 | 0.94-1.39 | 0.095 | | | | | | |
| I do not need vaccines for diseases that are not common anymore | 1.11 | 0.91-1.35 | 0.317 | | | | | | |
| We do not necessary to take voluntary vaccination | | | | | | | | | |
| I do not take vaccine, if everyone around me is immunized | | | | | | | | | |
| It is easy to obtain correct information on immunization | | | | | | | | | |

| | | | |
|---|---|---|---|
| It is easy to understand why immunization is needed. | 0.79 | 0.57-1.08 | 0.137 |
| I have been able to accurately understand the vaccinations I have received | 0.80 | 0.57-1.12 | 0.190 |
